# Visual function and ocular status of children with hearing impairment in Oman: A case series

**DOI:** 10.4103/0301-4738.49400

**Published:** 2009

**Authors:** Rajiv Khandekar, Mohammed Al Fahdi, Bushra Al Jabri, Saleh Al Harby, Talat Abdulamgeed

**Affiliations:** Eye and Ear Health Care, Department of Non-communicable Diseases Control, Directorate General of Health Affairs, Ministry of Health, Oman; 1 Department of Ophthalmology, Al Nahdhah Hospital, Ministry of Health, Muscat, Oman

**Keywords:** Hearing impaired, low vision, rehabilitation, visual disabled

## Abstract

Visual functions of children with hearing disability were evaluated in a school of Muscat, Oman in 2006. Two hundred and twenty-three children were tested for near vision, distant vision, contrast sensitivity, color vision, field of vision, motion perception and crowding. Profound and severe hearing loss was noted in 161 and 63 students respectively. Thirty-five (81%) students with refractive error were using spectacles. Color vision and field of vision was defective in one student each. In 286 (64.1%) eyes, contrast sensitivity was defective. Abnormal contrast sensitivity was not associated with the severity of hearing loss [RR = 1.04 (95% CI 0.91 to 1.29)]. Children with hearing impairment should be assessed for visual functions. Refractive error and defect in contrast sensitivity were unusually high among these children. In addition to visual aids, we recommend environmental changes to improve illumination and contrast to improve the quality of life of such children with double disability.

Children with visual impairment and hearing impairment face difficulty in performing daily activities. Early identification and specific rehabilitative efforts will improve the quality of life of such children. Visual functions of children with hearing impairment have been evaluated.[1–3] We present a cross-sectional study of visual function among children with hearing impairment and recommend rehabilitative approaches for improving the quality of life of these children.

## Materials and Methods

Permissions of Ministries of health and education were obtained for this study in 2006.

Optometrists assessed children in the school with the help of teachers. Presenting visual acuity was assessed. Portable trial set and direct streak retinoscope were used to test refraction. Hypermetropes underwent cycloplegic refraction. Near vision was tested with ‘Lea’s symbol chart’ held at 40 cm distance from the spectacles, and LogMAR values were noted.[4] Visual acuity for distance was measured at 3 meters, testing each eye separately. If symbol in the top line could not be identified, the test was repeated at 1.5meters and visual acuity was adjusted accordingly. Low contrast 2.5% chart was used to test contrast sensitivity. If it was not possible, contrast flip chart was used. The failure to identify the symbol determined the lowest percentage of contrast one could recognize at one meter. Peripheral field of vision was tested with a portable arc perimeter and white objects of 5mm and 10mm. The color vision was tested using 15 D test. Near vision chart with Lea's symbol having 25% and 50% crowding of symbols were used. Students with defective visual functions were retested. Low vision expert reassessed their results and provided low vision aids.

Health records were reviewed to determine the severity of hearing impairment. These children undergo testing of hearing with pure tone audiometry annually. Profound hearing loss means unaided hearing threshold of 81dB or more in the better ear. If the hearing threshold is 61 to 80dB, the child has severe hearing loss. The univariate analysis by parametric method was conducted using Statistical Package for Social Studies (SPSS -10). To associate the contrast sensitivity defects to the grades of hearing loss, we calculated relative risk and its 95% Confidence Interval. Different optical low-vision aids were tried and if appreciable improvement was noted, low-vision aids like hand magnifiers, stand magnifiers, telescopes, table lamp, filter glasses and felt pen were offered free of cost.

## Results

We examined 223 students (142 boys and 81 girls). ‘Five to 9’, ‘10 to 14’ and ‘≥ 15 years’ age group had 38, 85 and 100 students respectively. Profound hearing loss and severe hearing loss was reported in 62 (27.8%) and 161 (72.2%) students respectively.

Distant vision by the severity of hearing loss was analyzed [Table 1]. The risk of low vision among children with profound hearing loss was similar to those with severe hearing loss [RR= 1.45 (95% CI 0.7 to 2.97)]. Of the 43 students with refractive error, 37 (68%) students had spectacles.

**Table 1:** Visual status in better eye of persons with hearing impairment

		Students with profound hearing loss		Students with severe hearing loss	
	**Distant vision Log Values**	#	%		
Normal	0	46	74.2	99	61.5
	0.1	4	6.5	31	19.3
	0.2	2	3.2	13	8.1
	**Total**	**52**		**143**	
Low vision	0.3	3	4.8	9	5.6
	0.4	1	1.6	4	2.5
	0.5	3	4.8	1	0.6
	0.6	0	0.0	0	0.0
	0.7	1	1.6	2	1.2
	0.8	2	3.2	0	0.0
	0.9	0	0.0	0	0.0
	1.0	0	0.0	2	1.2
	**Low Vision**	**10**		**18**	
	**Total**	**62**		**161**	

One student each had defective field of vision and color blindness. The near vision acuity was defective in 29 (6.5%) eyes of 15 students. Near vision test for six eyes was not possible. Of 223 students, test for crowding phenomenon could not be carried out for four students. It was within normal range in 203 (91%) students and defective in 16 (7.2%) students.

If the difference was more than three lines in charts with 2.5% and 100% contrast symbols, the contrast sensitivity in that eye was defective. We evaluated 446 eyes of 223 students. Contrast sensitivity was normal in 129 (29%), but it was defective in 286 (64.1%) eyes. The contrast sensitivity was not associated to the severity of hearing loss [RR = 1.05 (95% CI 0.91 to 1.20)].

## Discussion

In this study we noted different types of visual function derangements in children with hearing impairment. Although we did not find an association of visual function to different grades of hearing impairment, such assessment could address at least one disability more effectively. A person with defective hearing and visual functions will face more difficulties in daily living.[5] These sensory impairments also affect the psychology of the person.[6] Hence visual and hearing impairment should be detected at the earliest and special care should be given.

Prevalence of hearing and visual function impairment in institutionalized population was much higher than in the general Dutch population.[7] Our study was conducted in a school for deaf children and had higher rates than that noted in the population survey.[8] Hence a holistic approach of assessing visual function of persons with other disabilities is recommended.

Spectacles were already provided to 68% of the needy before this assessment. The rest could be non-compliant children or they might have developed refractive error recently. More illumination is recommended to the children with lower contrast sensitivity. Students with near vision defects or with crowding problem could benefit with electronic gadgets to increase the space between the symbols/text. It would be crucial to review these children after corrective measures are applied to determine its impact on their quality of life.

We did not find an association of visual acuity and contrast sensitivity defects to grades of hearing impairment. Hearing and vision are affected significantly in the second and third decade of persons suffering from Usher's syndrome. However the type and degree of visual function and hearing impairment could vary in early ages.[9] Perhaps detailed examination using this test could identify preclinical stages of retinitis pigmentosa.[10]

All children with hearing loss are admitted to one school in Oman. It is possible that children, especially girls from far off places might not attend this school. Hence results should be extrapolated with caution.

We identified areas of visual function that are more likely to be compromised in hearing impaired. Proactive initiatives are needed to identify them as early as possible so that we can guide parents and teachers in altering the strategies for rehabilitating such individuals.
